# Mitochondrial division inhibitor-1 is neuroprotective in the A53T-α-synuclein rat model of Parkinson’s disease

**DOI:** 10.1038/s41598-017-07181-0

**Published:** 2017-08-08

**Authors:** Simone Bido, Federico N. Soria, Rebecca Z. Fan, Erwan Bezard, Kim Tieu

**Affiliations:** 1 University of Bordeaux, Institut des Maladies Neurodégénératives, UMR 5293 Bordeaux, France; 2 CNRS, Institut des Maladies Neurodégénératives, UMR 5293 Bordeaux, France; 3 Plymouth University Peninsula Schools of Medicine and Dentistry, Plymouth, United Kingdom; 4 Present Address: Florida International University, Miami, Florida USA

## Abstract

Alpha-synuclein (α-syn) is involved in both familial and sporadic Parkinson’s disease (PD). One of the proposed pathogenic mechanisms of α-syn mutations is mitochondrial dysfunction. However, it is not entirely clear the impact of impaired mitochondrial dynamics induced by α-syn on neurodegeneration and whether targeting this pathway has therapeutic potential. In this study we evaluated whether inhibition of mitochondrial fission is neuroprotective against α-syn overexpression *in vivo*. To accomplish this goal, we overexpressed human A53T-α- synuclein (hA53T-α-syn) in the rat nigrostriatal pathway, with or without treatment using the small molecule Mitochondrial Division Inhibitor-1 (mdivi-1), a putative inhibitor of the mitochondrial fission Dynamin-Related Protein-1 (Drp1). We show here that mdivi-1 reduced neurodegeneration, α-syn aggregates and normalized motor function. Mechanistically, mdivi-1 reduced mitochondrial fragmentation, mitochondrial dysfunction and oxidative stress. These *in vivo* results support the negative role of mutant α-syn in mitochondrial function and indicate that mdivi-1 has a high therapeutic potential for PD.

## Introduction

Parkinson’s disease (PD) is the second most common chronic neurodegenerative disorder after Alzheimer’s disease. It has been estimated that up to 10 million people worldwide are affected by PD. This number will drastically increase over time with our aging population. Disease-modifying therapies for this devastating disorder are urgently needed. We recently demonstrated that blocking Dynamin-Related Protein-1 (Drp1), a mitochondrial fission protein, rescued synaptic dysfunction and prevented cells death in the 1-methyl-4-phenyl-1,2,3,6-tetrahydropyridine (MPTP)-treated and *Pink1*-knockout mouse models of nigrostriatal dysfunction^1^, suggesting the potential clinical relevance to PD. The broad applicability of this therapeutic strategy and its translational potential in PD were investigated in the α-synuclein (α-syn) rat model in this study.

Available abundantly in presynaptic terminals, α-syn is a small 14 kDa protein that has received significant attention since it was discovered twenty years ago as the first genetic mutation in PD^2–5^. Recent genome-wide association studies have identified *SNCA* (that encodes α-syn) as a major gene associated with sporadic PD^6–8^. Identifying pathogenic mechanisms and effective therapies for α-syn neurotoxicity is thus relevant to familial, sporadic PD and other synucleinopathies. Although the exact physiological function of α-syn is still incompletely understood, missense mutations (A53T, A30P, E46K, H50Q and G51D) or gene multiplication mutations leading to higher levels of wild type α-syn cause autosomal-dominant PD^2–5, 9, 10^. α-syn is natively unfolded in solution^11^, but it has a propensity to form aggregates under various pathological conditions^12^. The aggregated and insoluble fibrillar form of α-syn constitutes a major component of the intracellular proteinaceous inclusions called Lewy bodies. Many interrelated pathogenic mechanisms of α-syn mutations have been proposed^13–15^, including mitochondrial dysfunction. Relevant to the present study, α-syn has been reported in recent years to induce severe mitochondrial fragmentation both *in vitro* and *in vivo*, although it is still a topic of debate whether this defect is a result of increased fission, impaired fusion or both, or reduced connectivity between mitochondria and endoplasmic reticulum^16–20^. However, the clinical relevance of targeting mitochondrial dynamics has not been determined in α-syn animal models.

Mitochondrial dynamics are controlled by fission and fusion proteins. Fusion requires the coordination of both the inner (IMM) and the outer (OMM) mitochondrial membranes. The OMM proteins Mitofusin 1 & 2 (Mfn1/2) coordinate with the IMM Optic Atrophy-1 (Opa1) to join the membrane. Mitochondrial Fission Factor (Mff), Fission-1 (Fis1), as well as Mitochondrial Dynamics Proteins of 49 and 51 kDa (MiD49 and MiD51, respectively) are anchored to the OMM where they recruit cytosolic Dynamin-related protein-1 (Drp1), which then oligomerizes and forms a ring-like structure around the mitochondria to constrict and split them into smaller ones^21^. Because Drp1 can bind to multiple fission proteins to sever mitochondria, directly targeting Drp1 is more effective to block mitochondrial fission. Furthermore, the translational potential of this target is enhanced by the availability of the small molecule inhibitor.

Mitochondrial division inhibitor (mdivi-1) was first identified as an inhibitor of mitochondrial division in yeast screens of approximately 23,000 compounds^22^. Its protective effects through blocking mitochondrial fission have been reported in cultured cells^23–28^ and mouse models^1, 26, 29–33^ of a wide range of diseases. We recently characterized its brain and plasma kinetics and demonstrated that it was protective in the *Pink1*-knockout and MPTP-treated mouse models of PD^1^. We report in this study that mdivi-1 is effective against hA53T-α-syn-induced neurodegeneration, protein aggregation, mitochondrial abnormalities, oxidative stress and motor impairment in rats.

## Results

### Mdivi-1 prevents motor function deficits and neurodegeneration

**Figure 1:**
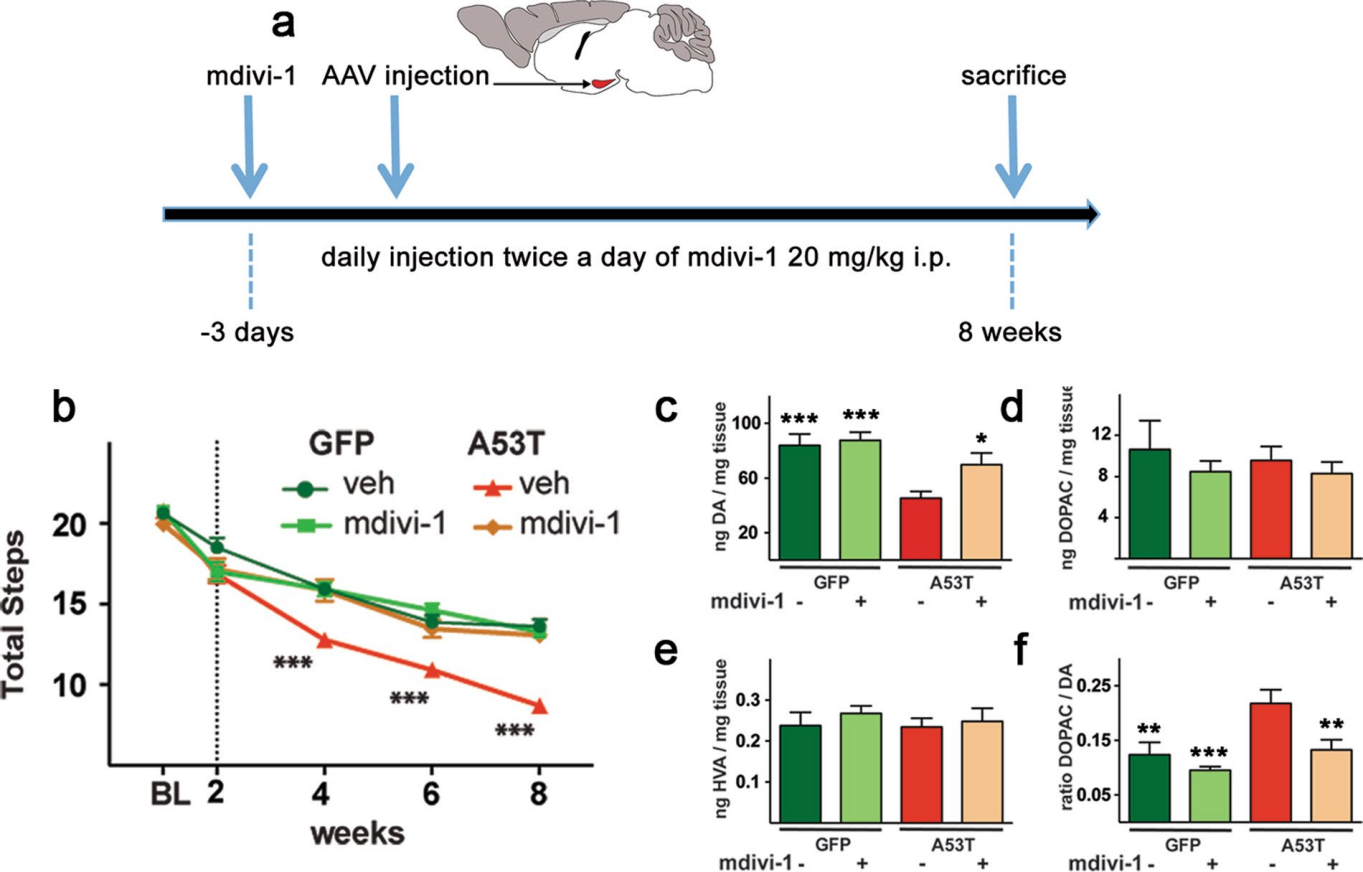
Mdivi-1 preserves motor function, dopamine levels and dopamine turnover rate against hA53T-α-syn-induced neurotoxicity. (**a**) Schematic illustration of the experimental design. (**b**) Sprague Dawley rats (6 weeks old) were assessed for locomotor function using stepping test. Afterwards, these animals were either injected with mdivi-1 (20 mg/kg, i.p) or vehicle control twice daily for the whole duration of the experiment. Three days after the initiation of mdivi-1 treatment, animals were stereotaxically injected with either AAV- hA53T-α-syn or AAV-GFPdegron control. Motor function was assessed every second week. Mdivi-1 treatment prevented motor deficits induced by hA53T-α-syn. HPLC was used to quantify total striatal dopamine (DA) content (**c**) as well as DA metabolites, (**d**) 3,4-Dihydroxyphenylacetic acid (DOPAC) and (**e**) Homovanillic acid (HVA). hA53T-α-syn induced significant DA depletion and increased DA turnover as evidenced by the ratio of DOPAC/DA (**f**). Mdivi-1 reduced this high turnover rate of DA (**e**) and restored the normal striatal level of DA (**c**). Mdivi-1 had no effect when injected in control animals (GFP). (**b**) Values are means ± SEM for 7–8 rats and were analyzed by 2-way ANOVA. (time: *F*_*4,135*_ = 224.5 p < 0.001; treatment: *F*_*3,135*_ = 32.81 p < 0.001; time x treatment: *F*_*12,135*_ = 5.651 p < 0.001) followed by Tukey multiple comparison test (p < 0.001 versus hA53T-α-syn vehicle injected animals). Data from neurochemistry (**c**–**f**) were analysed using two-way ANOVA (AAV injection: *F*_*1,26*_ = 10.57 p = 0.003; treatment: *F*_*1,26*_ = 7.916 p = 0.009; AAV injection x treatment: *F*_*1,26*_ = 1.984 p = 0.1708) followed by Tukey multiple comparison test (*p < 0.05; **p < 0.01; ***p < 0.001 compared to A53T vehicle).

We previously demonstrated that blocking mitochondrial fission, using whether rAAV-gene transfer technology or mdivi-1 to block Drp1 function, reduced neurodegeneration and synaptic dysfunction in MPTP-treated and *Pink1*-null mice^1^. However, due to the inherent limitations of these models, it was not feasible to assess motor function in those animals. In our newly developed and characterized virally induced human A53T-α-synuclein (hA53T-α-syn) rat model, there is progressive motor impairment and neurodegeneration^34^. Combined with the significant role of α-syn in PD, this model is highly suitable for testing the translational potential of mdivi-1. As we previously reported^34^, four weeks after receiving stereotaxic injection of hA53T-α-syn into the substantia nigra pars compacta (SNc, Fig. 1a), rats exhibited progressive and severe motor impairment as compared to the control group that received rAAV-GFP (Fig. 1b). Twice daily intraperitoneal (i.p.) injection of mdivi-1 [20 mg/kg, a dosage regimen that we previously characterized^1^], however, completely normalized this abnormality – even up to the 8 week-end point of this study (Fig. 1b). At the end of this behavioral study, animals were euthanized and further processed for biochemical and neuropathological alterations.

To correlate the change in striatal dopamine (DA) to motor function, we used High Performance Liquid Chromatography (HPLC) to quantify striatal DA content in these animals (Fig. 1c–f). Consistent with the observed motor function, severe DA depletion was detected after 8 weeks of expressing hA53T-α-syn (Fig. 1c). Other metabolites of DA were not affected (Fig. 1d,e). hA53T-α-syn also accelerates the turnover rate of DA (Fig. 1f). Mdivi-1 prevented the reduction of DA (Fig. 1c) and its high turnover rate (Fig. 1f) induced by hA53T-α-syn. Of note, mdivi-1 did not affect the total levels of DA and its metabolites in the GFP control animals, suggesting that blocking Drp1 does not have a detectable detrimental effect on healthy neurons.

**Figure 2:**
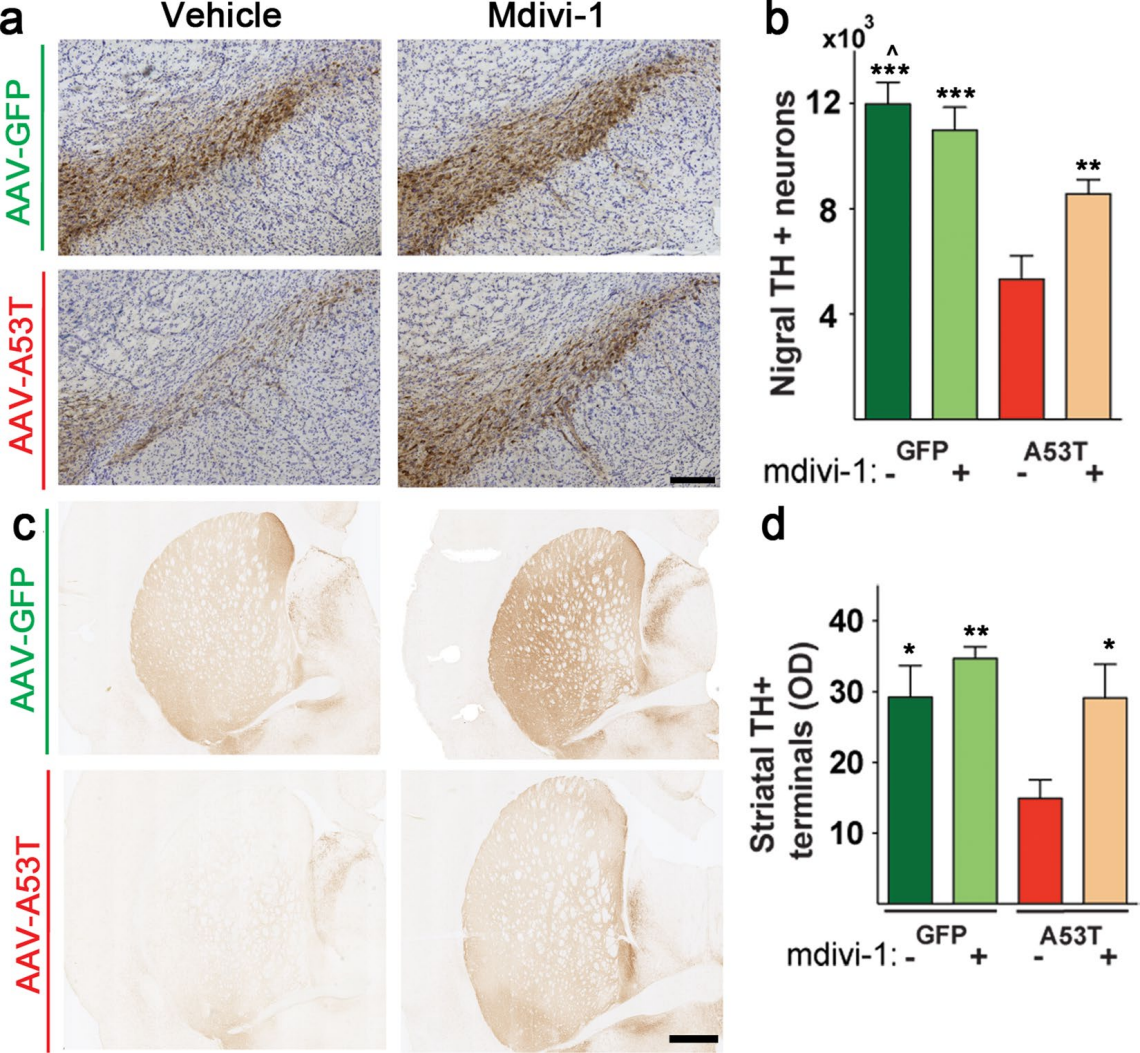
Mdivi-1 attenuates nigrostriatal degeneration induced by hA53T-α-syn. (**a**) Representative pictures of TH immuno-staining in SNc, scale bar 200 μm. (**b**) Stereological quantification in SNc showing a decrease in the total number of TH positive neurons in animals expressing hA53T-α-syn. The decrease was prevented in A53T rats injected with mdivi-1. (**c**) Representative pictures of TH staining in striatum. Scale bar 1 mm. (**d**) Optical density of TH positive fibers was reduced in rat expressing hA53T-α-syn compared to GFP and preserved in mdivi-1-treated A53T animals. (**b,d**) Values are means ± SEM of 7–8 rats analysed using two-way ANOVA (**b)**, AAV injection: *F*_*1,26*_ = 43.79 p < 0.001; treatment: *F*_*1,26*_ = 3.919 p = 0.0584; AAV injection x treatment: *F*_*1,26*_ = 11.12 p = 0.0026; (**d**) AAV injection: *F*_*1,27*_ = 7.83 p = 0.009; treatment: *F*_*1,27*_ = 7.664 p = 0.0101; AAV injection x treatment: *F*_*1,27*_ = 1.504 p = 0.23) followed by Tukey multiple comparison test (*p < 0.05; **p < 0.01; ***p < 0.001 to A53T vehicle, ^p < 0.05 to A53T mdivi-1).

Next, we performed stereological cell counting for accurate measure of the population of nigral dopaminergic neurons in these animals. Density of striatal dopaminergic fibers was also quantified. Consistent with the alterations in motor function and striatal DA content, hA53T-α-syn induced a dramatic loss of nigral dopaminergic neurons (Fig. 2a,b) and their striatal terminals (Fig. 2c,d). Mdivi-1 treatment conferred a significant protection in this nigrostriatal pathway. Together, these data strongly support the translational value of using mdivi-1 to reduce neurodegeneration and associated motor function deficits in PD.

### Mdivi-1 reduces proteinase K-resistant and phosphorylated α-synuclein

**Figure 3:**
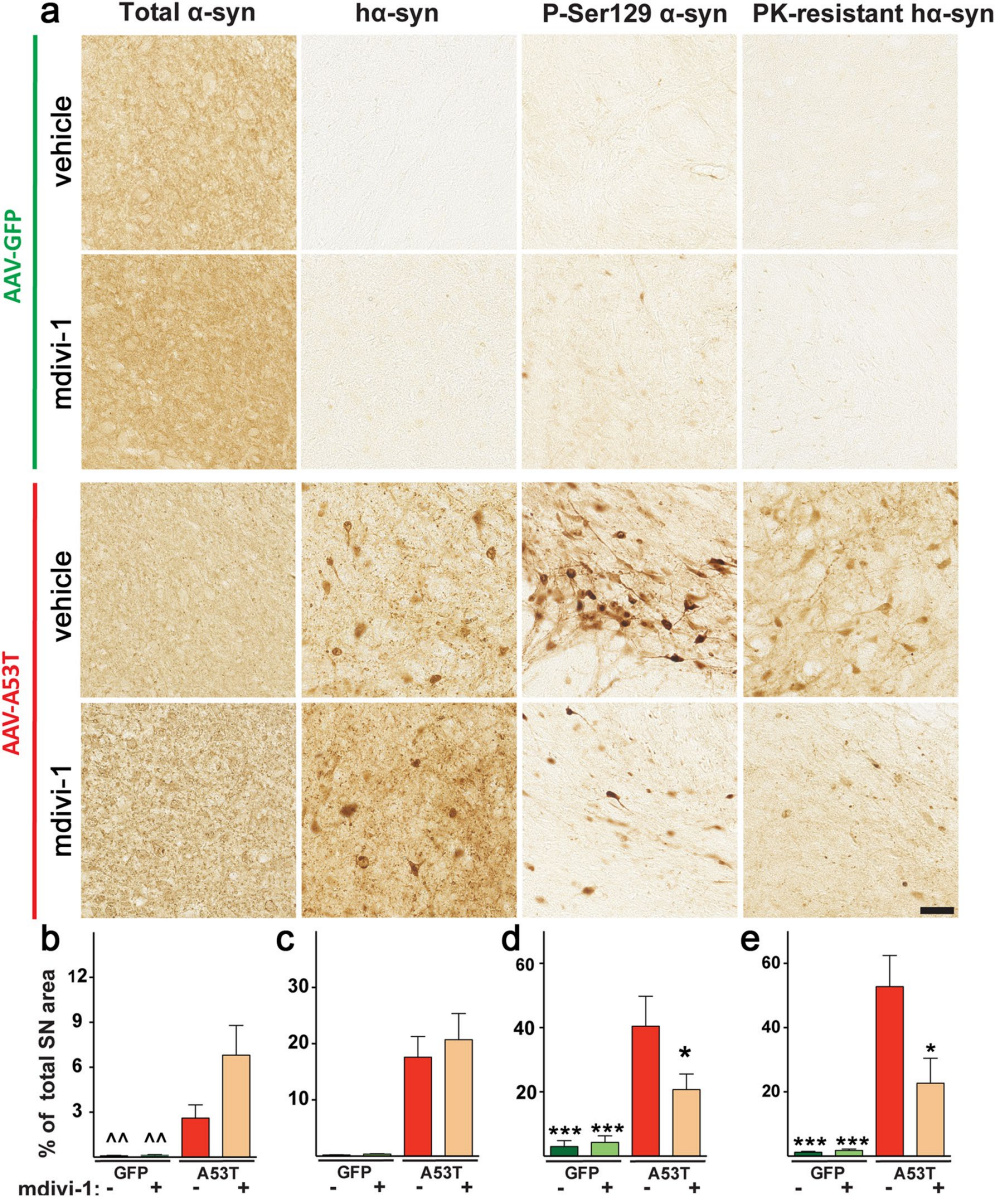
Mdivi-1-mediated neuroprotection is accompanied by a decrease in phospho- and PK-resistant α-syn. (**a**) Representative pictures of α-syn immuno-staining in SN of AAV-hA53T-α-syn and AAV-GFP transduced rats. From left to right: total (endogenous rat α-syn+ human A53T-α-syn), human A53T, phosphorylated and PK-resistant A53T α-syn. Scale bar 50 μm. (**b**) When quantified, the expression of total α-syn is higher in rats injected with hA53T-α-syn and increases further after mdivi-1 treatment, suggesting mdivi-1 preserved more dopaminergic structures. (**c**) As expected, higher levels of human α-syn were detectable in animals with hA53T-α-syn transduction. Mdivi-1 did not affect the total levels of this protein. (**d**) The level of phosphorylated α-syn was increased in A53T animals and, mdivi-1 treatment attenuated this upregulation. (**e**) Following PK treatment, a strong hA53T-α-syn immunoreactivity persisted in A53T animals. This signal was significantly reduced in A53T rats treated with mdivi-1. The quantification was carried out using an automated threshold in one slice per animal. Values are means ± SEM for 7–8 animals analysed by two-way ANOVA (**b**) AAV injection: *F*_*1,27*_ = 16.68 p < 0.001; treatment: *F*_*1,27*_ = 3.611 p = 0.0681; AAV injection x treatment: *F*_*1,27*_ = 3.476 p = 0.0732; (**d**) AAV injection: *F*_*1,27*_ = 22.69 p < 0.001; treatment: *F*_*1,27*_ = 3.176 p = 0.0859; AAV injection x treatment: *F*_*1,27*_ = 4.066 p = 0.0538; **e**, AAV injection: *F*_*1,27*_ = 31.45 p < 0.001; treatment: *F*_*1,27*_ = 5.217 p = 0.0305; AAV injection x treatment: *F*_*1,27*_ = 5.635 p = 0.025) followed by Tukey multiple comparison test (*p < 0.05; ***p < 0.001 to A53T vehicle, ^^p < 0.01 to A53T mdivi-1)

Aggregated and phosphorylated α-syn are prominent features in PD. Reducing such aggregation has long been proposed as a therapeutic strategy for this disease. To evaluate if mdivi-1 would reduce such modified α-syn, we performed immunohistochemistry on coronal sections containing *substantia nigra* (Fig. 3). In animals with hA53T-α-syn transduction, significantly increased levels in phosphorylated serine 129 α-syn and proteinase K-resistant α-syn were detected. However, mdivi-1 significant reduced the levels of these pathogenic α-syn forms, despite the fact that it did not change the overall levels of hA53T-α-syn. Taken together, these results indicate that mdivi-1 is capable of reducing protein aggregation and toxic phosphorylated α-syn.

### Mdivi-1 attenuates mitochondrial fragmentation

**Figure 4:**
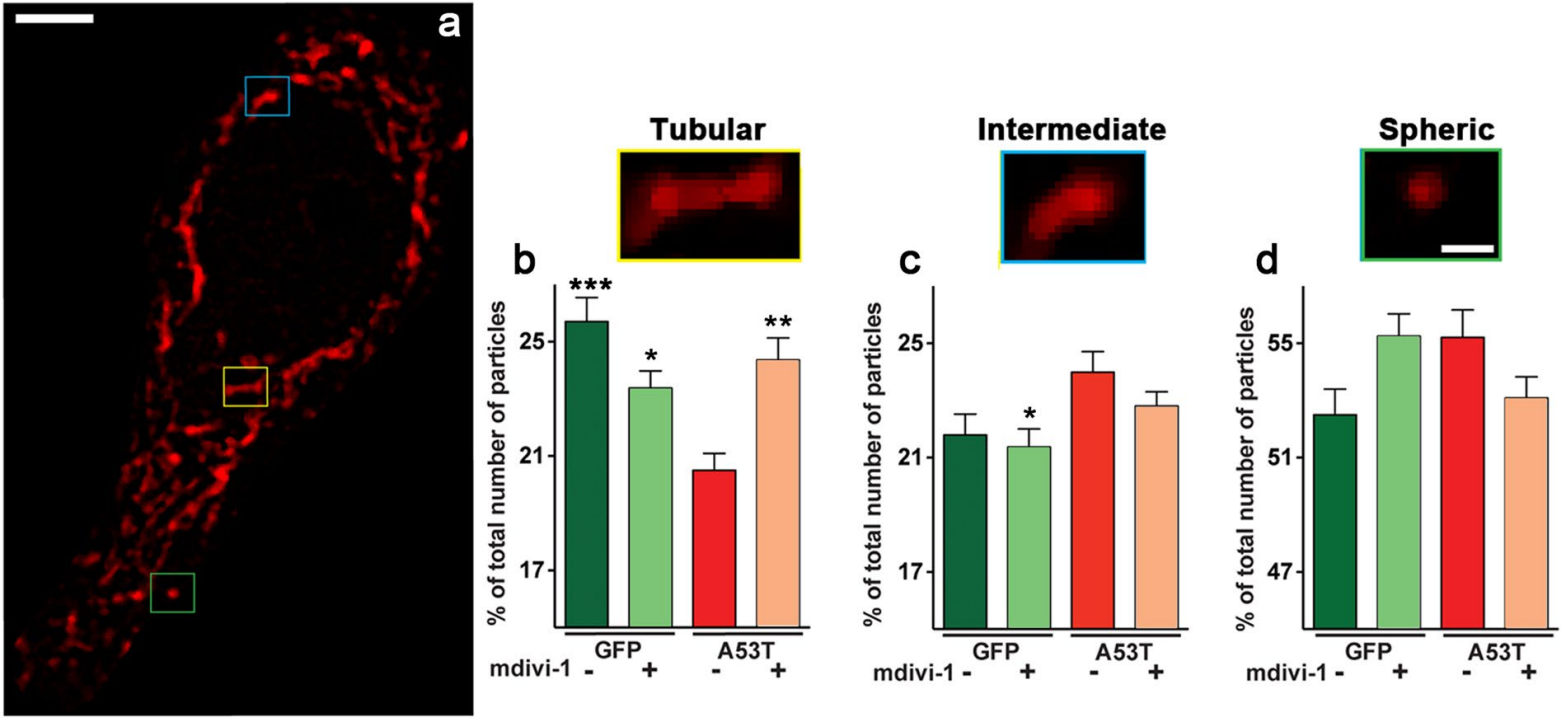
Mdivi-1 reduces mitochondrial fragmentation in dopaminergic neurons induced by hA53T-α-syn. Coronal midbrain sections containing SN of rats after 8 weeks of receiving AAV-hA53T-α-syn or AAV-GFPdegron with or without mdivi-1 treatment were immunostained with a mitochondrial marker HSP60 (red) and TH (not shown). Scale bar 5 μm. Different mitochondrial morphologies were categorized into three populations according to their shapes: tubular, intermediate and spheric (representative pictures bottom panel a, scale bar 1 μm). (**b,c,d**) Abundance in different mitochondria populations. Panel B shows a decrease in the relative number of tubular mitochondria in neurons of rats expressing hA53T-α-syn. This phenotype was reversed following mdivi-1 treatment. Fluorescent images were captured as single layer. Values are means ± SEM for 50–80 TH^+^ neurons per group. Statistical analysis was performed using two-way ANOVA (**b**) AAV injection: *F*_*1,246*_ = 8.144 p = 0.0047; treatment: *F*_*1,246*_ = 1.069 p = 0.3023; AAV injection x treatment: *F*_*1,246*_ = 17.47 p < 0.001;(**c**) AAV injection: *F*_*1,246*_ = 8.008 p = 0.005; treatment: *F*_*1,246*_ = 1.569 p = 0.2115; AAV injection x treatment: *F*_*1,246*_ = 0.3536 p = 0.5526) followed by Tukey multiple comparison test (*p < 0.05; **p < 0.01; ***p < 0.01 to A53T vehicle).

α-syn has been demonstrated to induce severe mitochondrial fragmentation in a number of cell culture studies^16–20^. In a recent *in vivo* study, mice with inducible expression of hA53T-α-syn in dopaminergic neurons exhibited severe mitochondrial fragmentation in a time dependent manner prior to axonal damage and progressive loss of these neurons^19^. However, to date, it is not clear whether mdivi-1 would prevent mitochondrial fragmentation in a hA53T-α-syn rodent model. To this end, we performed immunohistochemistry using Heat Shock Protein 60 (HSP60) as a mitochondrial marker and quantified mitochondrial morphology. Consistent with this previous animal study^19^, we also observed significantly fewer numbers of mitochondria with tubular morphology in rats that received rAAV9- hA53T-α-syn (Fig. 4). Mdivi-1 treatment provided protection against such morphological alterations in mitochondria. Although there was a trend of less tubular and more spherical mitochondria in the GFP control group, the difference is not statistically significant. Nevertheless, the absence of elongated mitochondria as we previously observed in mice^1^ was unexpected. Based on our previous extensive dose-response study of this molecule and overexpression of proteins that promote mitochondrial fusion (Mfn2), promote fission (Fis1) and blocking fission (Drp1-K38A)^23^, it is possible that the appearance of this spherical morphology was due to enhanced fission inhibition. In our previous study, using rat dopaminergic neuronal cells, we observed that mdivi-1 lost the ability to promote mitochondrial elongation in some cells at increasing concentrations. High levels of expression of Mfn2 and Drp1-K38A produced similar effects^23^. Together, these results suggest that a gene-dose effect of promoting mitochondrial elongation is an inverted U-shape response. Further supporting this idea, studies from other laboratories also observed smaller/fragmented mitochondria in cells with overexpression of Opa1^35^ and Mfn2^36^. Of importance in the present study is that mdivi-1 prevented mitochondrial fragmentation induced by α-syn-A53T.

### Mdivi-1 improves mitochondrial function in striatal synaptosomes

**Figure 5:**
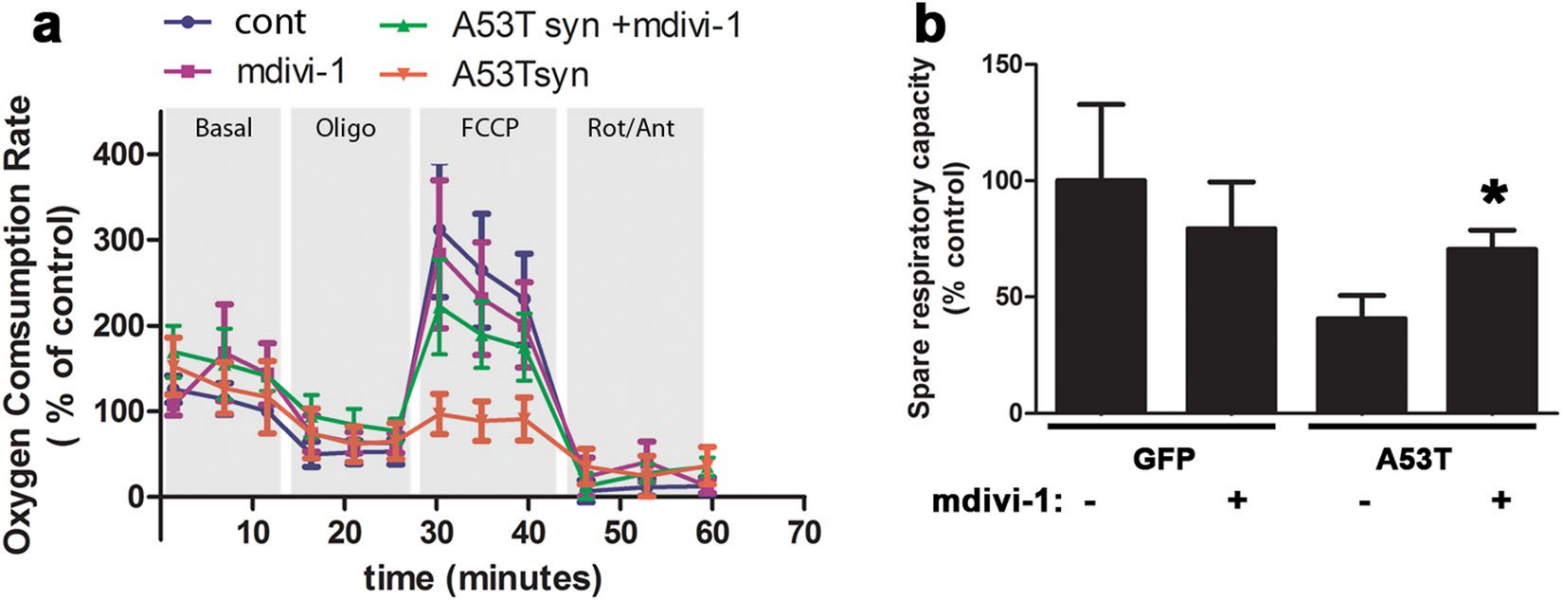
Mdivi-1 attenuates mitochondrial dysfunction in isolated synaptosomes. Mitochondrial function in striatal synaptosomes was assessed by measuring oxygen consumption rate (OCR) using the XFe96 Extracellular Flux Analyzer. (**a**) Sequential injections of oligomycin (to inhibit oxygen consumption mediated by ATP synthase), FCCP (an uncoupler to induce maximal OCR), Rotenone/Antimycin (to inhibit complex I and III, respectively). Values obtained from (**a**) were then used to calculate Spare Respiratory Capacity as a % = (Maximal Respiration)/(Basal Respiration) × 100 (**b**). Data represent means ± SEM of 5 five animals per group (except for the GFP+ mdivi-1 group, n = 3), Statistical analysis was performed using two-way ANOVA (AAV injection: *F*_*1,14*_ = 26.12 p < 0.001; treatment: *F*_*1,14*_ = 4.07 p = 0.063; AAV injection x treatment: *F*_*1,14*_ = 1.19 p = 0.294) followed by Tukey multiple comparison test (*p < 0.05 to A53T vehicle).

α-syn has been demonstrated in rodent models to reduce striatal presynaptic DA release^19, 37–41^ and impair mitochondrial respiration in the striatum^42^. Together, these observations are consistent with the critical role of mitochondria in synaptic function. To address whether blocking Drp1 improved mitochondrial function in the present study, we isolated synaptosomes from the striatum of rats with hA53T-α-syn or GFP with or without mdivi-1 treatment and measured mitochondrial function using XFe96 Extracellular Flux Analyzer as described^43^. This technology allows measurement of mitochondrial respiration in relatively small quantity and thus facilitates the assessment in specific brain regions. As seen in Fig. 5, one week after gene delivery (an early time point that induces about 50% cell death)^34^, hA53T-α-syn significantly impaired maximal rate of mitochondrial respiration as compared to the control group, resulting in reduced spare respiratory capacity (Fig. 5b). These results are consistent with a previous observation using human induced pluripotent stem cell (hiPSC) with hA53T-α-syn^44^. Spare respiratory capacity is the ability of mitochondria to provide substrate supply and electron transport to response to an increase in energy demand. A reduction in spare respiratory capacity leads to energy crisis when energy demand exceeds the supply ability of mitochondria. Indeed, spare respiratory capacity has been considered as a major factor that defines the survival of the neuron^43^. Mdivi-1 significantly improved spare respiratory capacity in animals with hA53T-α-syn.

### Mdivi-1 prevents oxidative stress

**Figure 6:**
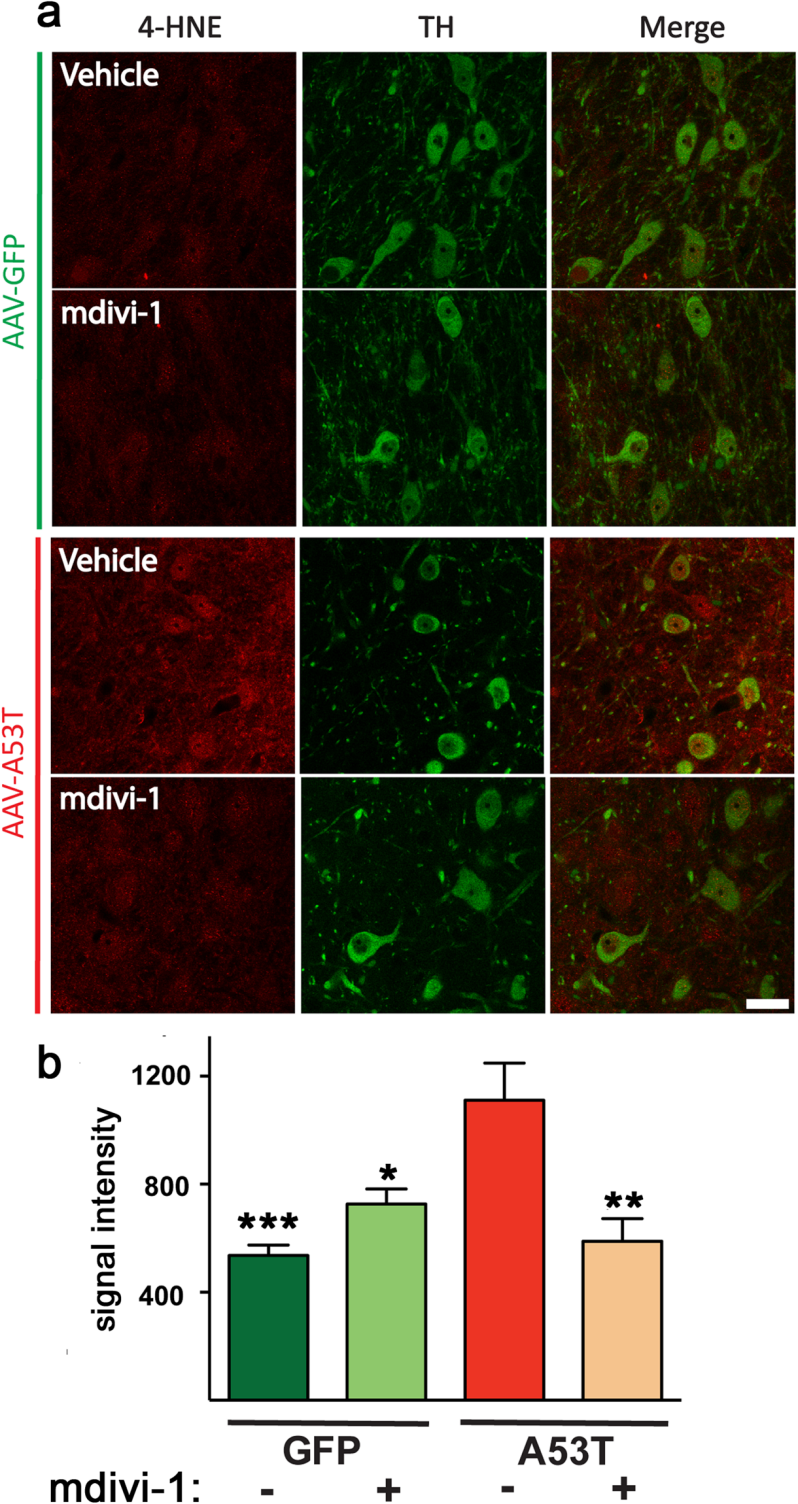
Mdivi-1 decreases ROS-mediated lipid oxidation in A53T animals. (**a**) Representative pictures of 4-HNE staining (red) measured in TH positive neurons (green). Scale bar 30 μm. (**b**) Dopaminergic cells of animals expressing hA53T-α-syn display an increase of lipid oxidation that turned back to basal levels in the presence of mdivi-1 treatment. Quantification was carried out measuring the levels of grey of 4-HNE staining in 16-bit images. Fluorescent images are captured as single layer. Values are means ± SEM for 7–8 rats (6 cells are analysed for each rat) and were analysed by two-way ANOVA (AAV injection: *F*_*1,27*_ = 5.196 p = 0.0308; treatment: *F*_*1,27*_ = 4.014 p = 0.0552; AAV injection x treatment: *F*_*1,27*_ = 16.91 p < 0.001) followed by Tukey multiple comparison test (*p < 0.05; **p < 0.01; ***p < 0.001 to A53T vehicle).

It is well-established that mitochondrial fragmentation and dysfunction generate oxidative stress. We asked whether oxidative stress also occurred in our animal model and if so, whether mdivi-1 would reduce it. To address this question, we performed immunohistochemistry to detect 4-hydroxy-2-nonenal (4-HNE), a major product generated from lipid peroxidation as a result of free radical attack. Eight weeks after AAV-hA53T-α-syn injection in rats, a high level of 4-HNE in nigral dopaminergic neurons was evident as compared to those transduced with AAV-GFP. Consistent with the effect of mdivi-1 on preserving mitochondrial morphology and function, mdivi-1 blocked the production of 4-HNE in the group of animals that received hA53T-α-syn (Fig. 6).

## Discussion

As we discussed in our recent reviews^13, 14^, misfolded α-syn (whether as a result of mutations, exposure to environmental toxins or infection) induces cellular dysfunction and neurodegeneration through several distinct but non-mutually exclusive mechanisms. For example, because of its strong propensity to bind to membranes, α-syn localizes to organelles such as lysosomes, endoplasmic reticulum and mitochondria. Indeed, it has been well-reported that α-syn impairs the function of these organelles. Combined with its ability to inhibit the ubiquitin-proteosomal and autophagic pathways, protein aggregation is a common observation in α-syn associated toxicity. In addition to these cell-autonomous mechanisms, α-syn also activates microglia and induces neuroinflammation, resulting in a non-cell autonomous-mediated toxicity. With such multiple pathogenic mechanisms, various therapeutic strategies have been developed over years to combat α-syn associated pathologies^13^. In the present study, we addressed the critical question of whether protecting mitochondrial integrity and function would be sufficient to attenuate α-syn-induced neurotoxicity *in vivo*.

Using our well-characterized virally induced hA53T-α-syn rat model^34^, we demonstrated that the putative Drp1 inhibitor mdivi-1 is highly effective in reducing dopaminergic neurodegeneration and motor dysfunction. At biochemical and cellular levels, mdivi-1 increased striatal DA content, reduced protein aggregation and oxidative stress, as well as reduced mitochondrial fragmentation and improved mitochondrial function. Altogether these results support the negative impact of α-syn on mitochondria and that blocking mitochondrial fission is protective against α-syn neurotoxicity. The results of our study are consistent with the role of α-syn in causing mitochondrial dysfunction in other *in vivo* studies. For example, Chesselet *et al*. demonstrated that mice with global overexpression of human wild type α-syn in the brain using the Thy1 promoter exhibited age dependent accumulation of α-syn in mitochondria in the nigrostriatal dopaminergic pathway, impaired electron transport chain function and enhanced oxidative stress^42^. Recently, Greenamyre’s laboratory reported that specific forms of post-translationally modified α-syn bind with high affinity to the mitochondrial receptor TOM20, resulting in mitochondrial dysfunction and production of reactive oxygen species^45^. From the Zhuang’s group, inducible hA53T-α-syn mice exhibited severe mitochondrial fragmentation that preceded dopaminergic neurodegeneration and other pathologies^19^_._ Using double immunogold-transmission electron microscopy, presence of the transgenic α-syn protein in mitochondria was clearly identified in these mutant mice. We also observed mitochondrial fragmentation in our present study using rAAV-hA53T-α-syn rat model. More importantly, for the first time, we demonstrated that blocking *in vivo* application of mdivi-1 is highly protective against hA53T-α-syn-induced neurodegeneration and other associated pathologies. The observation of mdivi-1 reducing accumulation of proteinase K-resistant and phosphorylated Ser129-α-syn is potentially significant. Additional studies are required to elucidate the mechanism of these effects. However, we hypothesize that the mechanism is mediated, at least in part, by improving mitochondrial function and reducing oxidative stress, because the autophagic and ubiquitin proteasomal pathways are energy dependent and sensitive to oxidative stress. A recent study has similarly reported that mdivi-1 reduced the accumulation of amyloid-beta plaque in double transgenic mice APP/PS1 model of Alzheimer’s disease^33^.

Manipulating mitochondrial fission/fusion has been considered as a potential novel mitochondrial therapy in recent years^46–48^. Within this context, blocking Drp1 is the most highly pursued strategy, partly because of the availability of a putative inhibitor (mdivi-1). Other investigators have demonstrated that blocking Drp1 function is protective in PD cell culture models of PINK1^23^, LRRK2^49, 50^ or VPS35^26^ mutations, and of rotenone^51^, MPP^+^^52, 53^ or 6-hydroxydopamine^54^ neurotoxins, as well as in *Pink1* ^*−/−*^ and MPTP-treated mice^1^. The protective role of blocking Drp1 in α-syn cell culture models is not quite definitive. Although some studies demonstrated that blocking Drp1 protected mitochondria from α-syn, others show that mitochondrial fragmentation induced by α-syn is Drp1 independent^16–18, 20, 55^. As often, conflicting data in cell culture models could be difficult to interpret due to different cell systems and experimental conditions. We believe that evaluations of the translational values of blocking Drp1 should extend beyond *in vitro* mitochondrial morphology and function, and that it should be conducted in mammalian animal models with brain pathologies and motor impairment as seen in PD. In this regard, the present study has provided some insights.

One potential concern of blocking mitochondrial fission as a therapeutic strategy is the possibility of developing side effects, because a balance of fission and fusion is necessary for the maintenance of neuronal function. This is an issue that will need to be considered if and when this treatment is to be conducted in clinical trials. Based on current literature, however, naive wild type mice are viable and no abnormal phenotypes are detectable up to ten weeks after the treatments of Drp1 inhibition, whether this is achieved by systemic injection of mdivi-1^1, 28–31, 33, 56–58^ or a peptide (P110-TAT)^59^ or by localized gene therapy^1^. Cytotoxicity of this molecule was not detectable at cellular, biochemical and functional levels, as shown in these publications and in the present study. Peripheral injections of mdivi-1 do not affect blood pressure, oxygen saturation, pH and blood cell counts^56^. Germline deletion of Drp1, however, induces embryonic lethality and degeneration of Purkinje neurons in mice^60–62^. Mice with conditional knockout of Drp1^63^ or Mfn2^64^ also indicate that nigrostriatal dopaminergic neurons are vulnerable to complete deletion of fission and fusion proteins. Interesting mice with heterozygous deletion of Drp1 have normal lifespan, phenotype, mitochondrial and synaptic structures^65^. Crossing these *Drp1* ^+/−^ mice with either the transgenic AβPP mice (Tg2576) or with Tau P301L transgenic mice reduced toxic soluble proteins and improved mitochondrial function in these animal models of Alzheimer’s disease^66, 67^. Together these studies indicate that there is a gene-dose effect of loss of Drp1 function on its associated negative impact on neuronal function and viability. Furthermore, partial Drp1 loss of function appears to be safe and sufficient to confer neuroprotection.

Mdivi-1 has been demonstrated independently by many laboratories to have striking protective effects in a wide-range of disease models both *in vitro* and *in vivo*. The interest in the translational potential of this molecule is therefore understandably high. However, recent studies have raised the question of the specific mechanism of action of mdivi-1. Initially discovered by Nunnari and colleagues, mdivi-1 was shown to be specific and potent inhibitor of GTPase activity of yeast Dnm1, a homolog of mammalian Drp1^22^. Subsequently, this small molecule was also demonstrated to block Drp1 GTPase activity in human recombinant Drp1^68^ and in mammalian neuronal cells^28^. GTPase activity of Drp1 is required for Drp1 oligomerization. Largely based on evidence from studies such as these ones, mdivi-1 has been considered in general as a Drp1 inhibitor. However, studies from the laboratory of Reddy showed that mdvi-1 also increased the levels of mitochondrial fusion proteins (Mfn1/2 and Opa1) and reduced the levels of Drp1 in neuronal cells^27, 28^. It has also been demonstrated *in vivo* to reduce the levels of phosphorylated Drp1-S616 induced by kainic acid in the mouse hippocampus^58^. Taken together, these studies indicate that mdivi-1 is capable of blocking mitochondrial fission and promoting mitochondrial fusion at both enzymatic and protein expression levels. It is worth noting that also using human recombinant Drp1, a recent study reported that mdivi-1 is a weak and non-specific inhibitor of Drp1 GTPase^69^. Instead, this molecule was reported to be a reversible inhibitor of complex I and ROS production generated via reverse electron transfer mechanism. More studies are clearly required to reconcile this discrepancy and explain why blocking complex I would confer protection observed in other studies. Rather than using recombinant Drp1, perhaps an intact mammalian cell system should be used to determine whether mdivi-1 would block Drp1 function either directly or indirectly. Taken together, it is clear so far that mdivi-1 confers striking protective effects across multiple disease models, but most likely not exclusively through Drp-1 inhibition.

In summary, the present study reports that mdivi-1 is highly effective in reducing neurodegeneration, motor dysfunction and accumulation of toxic α-syn, mitochondrial damage and oxidative stress in a rat α-syn model of PD. This study further highlights the translational potential of mdivi-1. In addition to the present study, this small molecule has been demonstrated to be beneficial in other rodent models of PD^1^, Huntington’s disease^28^, Alzheimer’s disease^33^, epilepsy^58^, renal^30^, cardiac^29^, brain ischemic damage^31, 70^, neuropathic pain^57^ and diabetes^32^. With such a striking neuroprotective property in a wide range of diseases, mdivi-1 and perhaps strategies of blocking Drp1 function hold great promise to novel therapies.

## Methods

### Antibodies

#### Primary

Mouse anti-TH (1:10000, 1:2000 for SNc and striatum respectively, clone LNC1, Millipore); mouse anti-α-synuclein (1:1000, BD Transduction Laboratories); mouse anti-human-α-synuclein (1:1000, clone LB509, Invitrogen); rabbit anti-α-synuclein-P-S129 (1:1000, Abcam ab59264); goat anti-HSP60 (1:20, Santa Cruz Biotechnology); Rabbit anti-4-HNE (1:200, Alpha Diagnostic).

#### Secondary

All the secondary antibodies used for the immunofluorescience staining are anti-IgG conjugated with Alexa Fluor^TM^ 488 or 568. For HRP immunohistochemistry labeling, the HRP Dako Envision Polymer^TM^ conjugated with the appropriate secondary antibody was used.

### Animals and experimental design

All experiments were performed in accordance with the European Union directive of September 22, 2010 (2010/63/EU) after approval by Institutional Animal Care and Use Committee of Bordeaux (CE50) under the license number 5012066-A, and in accordance with the provisions laid down by the UK Home Office under the Project Licence No. PPL 30/3088. Animals had free access to water and food. Veterinary care includes a full program for prevention of disease, daily observation and surveillance for animal health, appropriate methods of disease control, diagnosis, and treatment, appropriate methods of handling, restraint, anesthesia, analgesia and euthanasia as well as monitoring of surgical programs and post-surgical care. One week before surgery, Sprague Dawley rats (32 animals, 6 weeks old), purchased from Charles River, were tested for stepping test and sorted in order to generate 4 groups with no differences in the mean motor performance (p > 0.95). The 4 groups were then submitted to different treatments: 1) AAV-GFPdegron plus vehicle (GFP + veh), 2) AAV-GFPdegron plus mdivi-1 (GFP + mdivi-1), 3) AAV- hA53T-α-syn plus vehicle (A53T + veh) and 4) AAV- hA53T-α-syn plus mdivi-1 (A53T + mdivi-1).

### Mdivi-1 treatment

As previously described^1^, 3-(2,4-Dichloro-5-methoxyphenyl)-2-sulfanyl-4(3H)-quinazolinone (mdivi-1, Sequoia Research Products, UK) was dissolved in DMSO as a stock solution, which was then diluted with sterile 0.9% saline solution (1% DMSO final concentration), sonicated for 30 seconds and then promptly injected. Three days before stereotaxic injection of AAV- hA53T-α-syn or AAV-GFPdegron, animals were pre-treated with either mdivi-1 (20 mg/kg) or vehicle. This dosage was selected based on our previous study^1^, in which we characterized the *in vivo* pharmacokinetics and dose-response of mdivi-1. We found that with this dosage regimen, mdivi-1 conferred the most neuroprotective effects without detectable side-effects. The intraperitoneal (i.p) injections were performed twice a day (9 a.m.–18 p.m.) for eight weeks. During this period, every two weeks, motor performance was monitored with the stepping test. At the end of the treatment period animals were euthanized and brain collected for histological and neurochemical analysis. Brains were collected fresh and divided in three parts: bilateral mesencephalon and unilateral striatum was post fixed for 5 days in PFA 4% and then sectioned for histological studies, while another striatum was collected fresh, flash frozen and store at −80 °C for neurochemical analysis.

### Viral vector production

AAV9-GFPdegron and AAV9-hA53T-α-syn were produced by triple transfection into HEK-293T/17 cell line (ATCC, Teddington, UK) in polyethylenimine solution. After seventy-two hours cell were re-suspended with Tris lysis buffer (NaCl 150 mM, Tris-HCl 50 mM pH 8.5) and lysed using the freeze-thaw cycle procedure (−80 to +37 °C). The supernatant underwent to iodixanol gradient step purification (by centrifugation), and the fraction enriched in viral vector stocked at −80 °C. These procedure were well established and well described in our previous publications^34, 71^.

### Surgery procedure

Rats were bilaterally injected with AAV-GFPdegron or AAV- hA53T-α-syn (titer normalized to 6.9 × 10^13^ gcp/ml) as previously described^34^. Briefly, under isoflurane anesthesia, animals were sterotaxically injected with 2 μL of virus into the *substantia nigra pars compacta* (SNc) according to the following coordinates: AP = −4.9/−5.4, L = −2.2/−2, DV = −7.8 from bregma.

### Motor performance assessment

The stepping test was used to assess the forelimb akinesia as we recently described^34^. Rats were held and dragged sideways on a smooth surface at a constant speed for 0.9 m of distance and the number of adjusting steps counted. The performance was evaluated once every two weeks, three sessions over two consecutive days. The scores over the three sessions were averaged and the left and right backhand steps were pooled together.

### HPLC analysis

Fresh striatum collected after euthanasia was sonicated in HClO_4_ 0.1 M and the homogenate centrifuged at 4 °C for 30 min at 13000 rpm. 20 uL of supernatant was used for the HPLC analysis as previously described^72^. Monoamines were measured by coulometric detector (Coulochem II, ESA) coupled to a dual-electrode analytic cell (model 50110) with the potential of electrodes set at +350 and −270 mV. The samples were injected with a mobile phase containing NaH_2_PO_4_ 70 mM, methanol 7%, sodium octyl sulfate 100 mg/L, triethylamine 100 μL/L, EDTA 0.1 mM, into an HPLC Equisil column (C18, 150 × 4.5 mm, 5 μm)^72^. Retention times for noradrenaline, DOPAC, DA, HVA and serotonin were ~250, 420, 660, 1100, 1970 sec, respectively.

### Immunostaining

#### Tyrosine Hydroxylase

Free floating 50 μm-thick slices were rinsed in PBS and treated for one hour with a blocking solution containing BSA 2% and Triton × 100 0.3% in PBS. After being blocked, the tissue was incubated with the TH primary antibody diluted with a solution containing BSA 1%, Triton × 100 0.3% over night at room temperature. The slices were then incubated with the secondary antibody and finally revealed with peroxidase EnVision^TM^ system (DAKO). For the SNc slices the counter coloration with cresyl violet was performed. The slices were mounted in gelatin-coated slides and the coverslip sealed with Eukitt^TM^ mounting medium.

### Stereological counting

The unbiased stereological sampling method was used to quantify dopaminergic neurons in SNc as described in many occasions^34^ The cell counting was performed using Leica DM600 motorized microscope equipped with Mercator Pro Software (Explora Nova, La Rochelle, France). After SNc boundaries delimitation, TH positive (TH+) cells in SNc are on-line counted at 40X magnification over five 50 μm-thick sections for each brain, collected every 300 μm, encompassing the whole SNc. The optical fractionator stereological probe was then used to estimate the total number of TH+ neurons for the entire SNc volume. Considering the bilateral nature of our model, the data presented in this paper are the sum of neurons counted for both SNc.

### Striatal TH quantification

Images were taken with Nanozoomer 2.0 HT (Hamamatsu, Japan) at 20X magnification and analyzed with Image J (NIH, USA). The striatum boundaries were traced and optical density measured in terms of grey levels for 8-bit images as previously described^34^.

### α-synuclein immunostaining and quantification

For α-syn immunolabeling we applied the same protocol as described above for the TH staining as previously^73^. The slices treated with proteinase K (PK) were incubated for 10 minutes in a solution with 1 μg/mL of PK prior to any step and the tissue processed for the immunostaining. SNc pictures were captured with NanoZoomer using 20X objective and analyzed with Image J software. The quantification was carried out by measuring the percentage of the SNc area occupied by the stained surface using an automated threshold for all the images.

### Mitochondrial network staining *in vivo*

HSP60 immunostaining was used to characterize the mitochondrial phenotype in dopaminergic neurons of SNc. Free-floating coronal SNc sections of 50 µm thickness were rinsed ﻿in PBS, incubated for 10 min with H_2_O_2_ 3% and 10% methanol, then for 20 min with Triton × 100 2%. BSA 3% for 1 hour was used to saturate the unspecific binding site before the overnight incubation with primary antibody (diluted in a solution containing BSA 1% and Triton × 100 at room temperature. Following incubation, sections were rinsed three times for 10 min in PBS and incubated for 1 hour with the secondary antibody. The same protocol was used to co-stain the TH and phosphorylated α-syn. The triple staining was needed to limit the quantification to dopaminergic neurons (TH positive) expressing hα-syn (indicated by the accumulation of phospho-syn). Single layer pictures of rat SNc were taken with confocal microscope DM6000 TCS SP5 (Leica, Germany) using 63 X (1.4 NA) magnification.

### Mitochondrial network quantification *in vivo*

Every single neuron picture was analyzed through an Image J macro purposely designed for the quantification of the relative area, shape descriptors and number of mitochondrial particles occupying the cytoplasmic surface of the neuron. Moreover, the circularity index provided by the macro, allowed us to sort the entire mitochondria population fragments into three different groups based on their shape: 0.8 to 1 spheric; 0.5 to 0.79 intermediate; 0 to 0.49 tubular.

### 4-hydroxynonenal staining and quantification

TH and 4-HNE were co-stained following the same protocol used for the mitochondrial labeling *in vivo*. Single layer pictures were captured using confocal imaging at 40X magnification. We used a fixed laser power and AOTF levels for all the images. 16-bit pictures were then analyzed with Image J software measuring the level of grays of 4-HNE staining only in TH positive neurons.

### Synaptosomes isolation

Synaptosomes were isolated from Sprague Dawley adult rats based on a previous publication^43^ but with some modifications. Briefly, striata were quickly removed, rinsed with ice-cold sucrose buffer (320 mM sucrose, 1 mM EDTA, 0.25 mM dithiothreitol, pH 7.4) and then homogenized (10–12 strokes) in dounce glass homogenizer containing 1–1.5 mL sucrose buffer. The homogenates were then gently layered onto a discontinuous Percoll gradient (2.5 ml of 3%, 10% and 23% in sucrose buffer) in 10 mL centrifuge tubes, and centrifuged at 32,500 *g* for 10 min at 4 °C in a JA-25.50 fixed angle rotor in a Beckman Avanti J-26 X centrifuge. The band between 10% and 23% from striatum were collected for synaptosomes, pelleted and resuspended into ionic buffer (20 mM HEPES, 10 mM D-glucose, 1.2 mM Na2HPO4, 1 mM MgCl2, 5 mM), protein concentrations were measured using nano drop 2000.

### Mitochondrial function in striatal synaptosomes

Primary based on the method developed by David Nicoll’s laboratory^43^, striatal synaptosomes (2ug/well) were plated into an XF96 cell culture microplate (Seahorse Bioscience Inc.) which was previously sequentially pre-coated with 0.0033% (v/v) polyethyleneimine (Sigma-Aldrich) and Geltrex (Invitrogen). The plate was centrifuged at 3,400 *g* for 1 h at 4 °C in a Henttich Rotanta 460 R centrifuge to facilitate attachment. The ionic buffer was then replaced with 175 µL assay buffer (3.5 mM KCl, 120 mM NaCl, 1.3 mM CaCl2, 0.4 mM KH2PO4,1.2 mM Na2SO4, 15 mM D-glucose, 10 mM pyruvate, 0.4% (w/v) fatty acid-free bovine serum albumin, and 10 mM TES (N-[tris(hydroxymethyl)methyl]-2-aminoethanesulfonic acid), pH 7.4). The plate was incubated in a non-CO_2_ incubator at 37 °C for 10–15 min, then loaded into the XFe96 Extracellular Flux Analyzer (Seahorse Biosciences Inc.). Mitochondrial function in these synaptosomes, as indicated by oxygen consumption rate (OCR), was monitored in real-time throughout the assay. Different parameters of mitochondrial respiration were obtained with sequential injections of 2.5 μg/ml oligomycin, 4 μM FCCP and 2 μM Rotenone/Antimycin. Oxygen consumption rate data points represent the mean rates of each measurement cycle, which consisted of 30 s mixing time, 30 s waiting time, followed by 3 min of data acquisition. Basal respiration was measured before first injection (3 cycles), and 3 data points were obtained following each injection (12 data points in total).

### Statistics

All values are expressed as mean ± SEM. Differences between means were analysed using a two-way analysis of variance (ANOVA) followed by Tukey multiple comparison post-hoc test using GraphPad Prism v5.01. The null hypothesis was rejected when P-value was <0.05.
